# BRET‐Based Approaches for Ion Channels: Emerging Applications and Future Directions

**DOI:** 10.1002/ardp.70325

**Published:** 2026-09-03

**Authors:** Nadine Kock, Laura Heitzer, Joana Massa, Marvin Taterra, Marcel Bermúdez, Oliver Koch, Matthias Schiedel

**Affiliations:** ^1^ Institute for Pharmaceutical and Medical Chemistry, Faculty of Chemistry and Pharmacy University of Münster Münster Germany; ^2^ GRK 2515, Chemical biology of ion channels (Chembion) Universität Münster Münster Germany

**Keywords:** assay development, bioluminescence, high‐throughput screening, NanoBRET, target engagement

## Abstract

Bioluminescence resonance energy transfer (BRET) is a technique based on energy transfer between a luminescent enzyme and a fluorescent acceptor. Since its first description in 1999, BRET has been widely applied in chemical biology research and drug discovery. Today, it is regarded as one of the most versatile methods for investigating the dynamics of ligand–protein and protein–protein interactions, both in cell‐free systems and living cells. Owing to several advantages, including compatibility with high‐throughput screening, suitability for cellular target engagement studies, and the ability to monitor real‐time kinetics, BRET‐based binding assays have become indispensable tools, particularly in early‐stage drug discovery. Consequently, over the past decade these approaches have been extensively used for major drug target classes such as G protein‐coupled receptors (GPCRs), kinases, and proteases. Despite the high relevance of ion channels as drug targets—approximately one‐fifth of all approved drugs act on them—BRET‐based assays have so far played only a limited role in this field. Here, we summarize recent applications of BRET‐based methods for ion channels with a particular focus on approaches for studying ligand‐ion channel interactions, highlighting current challenges and, more importantly, the significant potential of this technology to advance ion channel–focused drug discovery.

## Graphical abstract

Bioluminescence resonance energy transfer (BRET) is a versatile method for studying ligand–protein interactions. Its compatibility with high‐throughput screening and real‐time kinetics has made it widely used in drug discovery. Here, we summarize recent applications of BRET‐based methods for studying ligand binding to ion channels, highlighting key challenges and the significant potential of this technology to advance ion‐channel drug discovery.

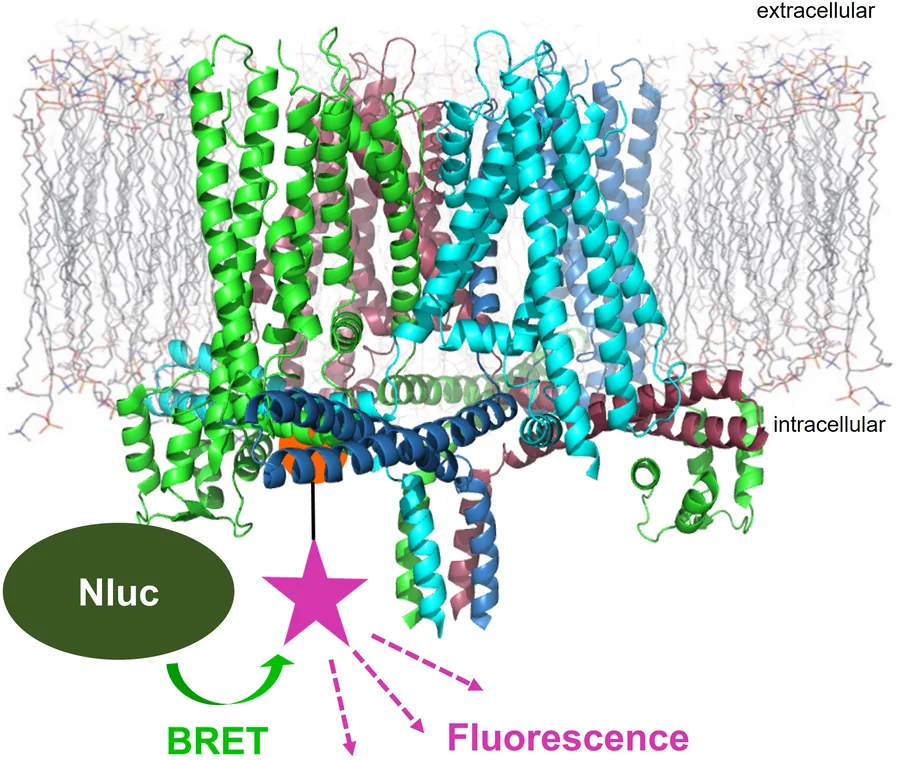

## 1 Introduction

Ion channels are integral membrane proteins that play a crucial role in a wide range of physiological processes, from nerve impulse transmission and heart function to immune regulation and hormone secretion [1, 2, 3, 4, 5]. These proteins control the flow of ions across cell membranes and are therefore fundamental to maintain cellular homeostasis and signal transduction [4, 5, 6]. Regarding their structure, ion channels are highly diverse and differ in the location of their C‐ and N‐ termini, the presence and size of cytosolic and/or extracellular domains, and the assembly as homo‐ or heteromers [7, 8, 9]. The vast array of different stimuli (*e.g*., voltage, small molecules, pressure, and temperature) results in an enormous physiological heterogeneity [10, 11, 12, 13]. Depending on their gating mechanism, ion channels can be categorized into voltage‐gated ion channels (*e.g*., Na_v_1), ligand‐gated ion channels (*e.g*., P2X2 channels), and temperature‐sensitive or mechanosensitive ion channels (*e.g*., transient receptor potential [TRP] and PIEZO channels) [14, 15, 16, 17, 18, 19, 20, 21]. Alternatively, ion channels can be categorized according to the specific ions they allow to pass, for example, as potassium‐, sodium‐, calcium‐, or chloride‐selective channels. Due to their central importance in numerous diseases such as neuropsychiatric disorders, cardiovascular and metabolic diseases, cancer, and immunological diseases, ion channels represent an attractive target class for the development of new drugs [5, 8, 11]. In fact, one‐fifth of all approved drugs act on ion channels, making them the second largest class of drug targets after GPCRs [4, 7, 22]. Currently, over 170 ion channel‐modulating drugs are approved (2024), and more than 60 are undergoing clinical trials (2024) [22]. This highlights the therapeutic relevance and potential of this protein family as important targets for the development of innovative drugs.

Understanding ligand binding to ion channels and the effects of this binding event on ion‐channel activity is crucial for the discovery of new drugs for this target class. A range of conventional techniques are employed to investigate these properties.

For measuring the activity, electrophysiological methods, such as patch‐clamp, are most commonly utilized since ion channels are involved in the change of the membrane potential due to ion flux. Patch‐clamp can be used in a whole‐cell manner or at specific membrane areas to measure the activity of one particular ion channel of interest, using a glass pipette that is connected to an electrode.

Therefore, this method provides high sensitivity and temporal resolution by directly measuring changes in ion currents resulting from ligand‐induced ion‐channel opening and closing, respectively. The downsides of this method are the time‐consuming training of new operators, which can take several months, and the limited assay throughput [23, 24, 25, 26]. To increase the accessibility of patch‐clamp for high‐throughput screenings (HTS), automated patch‐clamp (APC) systems such as Qpatch were developed, thereby matching the assay quality of manual patch‐clamp while substantially increasing throughput [24, 27, 28]. Besides electrophysiological methods, fluorescence‐based assays are implemented to monitor ion flux or membrane potential changes in a high‐throughput manner. To this end, fluorescent dyes that react to specific ion or voltage changes are incubated together with the cell. Depending on the fluorescent dye and the effect triggered by the addition of a ligand, the observed fluorescence signal is reduced or increased in the presence of an active ligand. Although fluorescence‐based assays are less precise than patch‐clamp methods, they are widely used for the initial screening of drug candidates due to their substantially higher throughput [4, 25, 29]. Overall, both electrophysiological and fluorescence‐based activity assays are able to provide key information about the change in ion‐channel activity as a consequence of ligand binding.

However, particularly in the early stages of drug discovery, determining the binding affinities and kinetics of newly identified ligands using suitable binding assays is essential for prioritizing the most promising candidates for further development [30, 31]. Importantly, binding assays for membrane proteins do not necessarily require an intact cellular environment and can often be performed using cell‐free preparations [32, 33], thereby simplifying assay implementation and facilitating experimental optimization. Moreover, binding assays enable the straightforward mapping of ligands to specific binding sites on the target protein, providing valuable information on orthosteric versus allosteric binding and enabling the characterization of distinct binding sites and mechanisms of action [34].

In this context, radioligand binding assays are widely used, also in the field of ion channels. Such assays enable the determination of ligand affinities for unlabeled ligands through displacement of radioactive tracers [35, 36, 37, 38]. However, isotopic assays are limited by their reliance on radioactive tracers, which entails several drawbacks, including stringent safety precautions, the need for specialized facilities and permits, high cost for the procurement of radiolabeled tracers, and expensive disposal of radioactive waste. In addition, they often involve laborious (heterogeneous) assay protocols with washing steps to remove the unbound radioligand fraction before assay readout [39, 40]. The latter is also the reason why radioligand binding assays are often not well suited for kinetic measurements with a continuous readout, the detection of low‐affinity binders, or cellular target engagement studies for intracellular binding sites [34]. Consequently, there remains a need for efficient, sensitive, and high‐throughput methods to comprehensively characterize binding interactions between ion channels and novel drug candidates.

**Figure 1:**
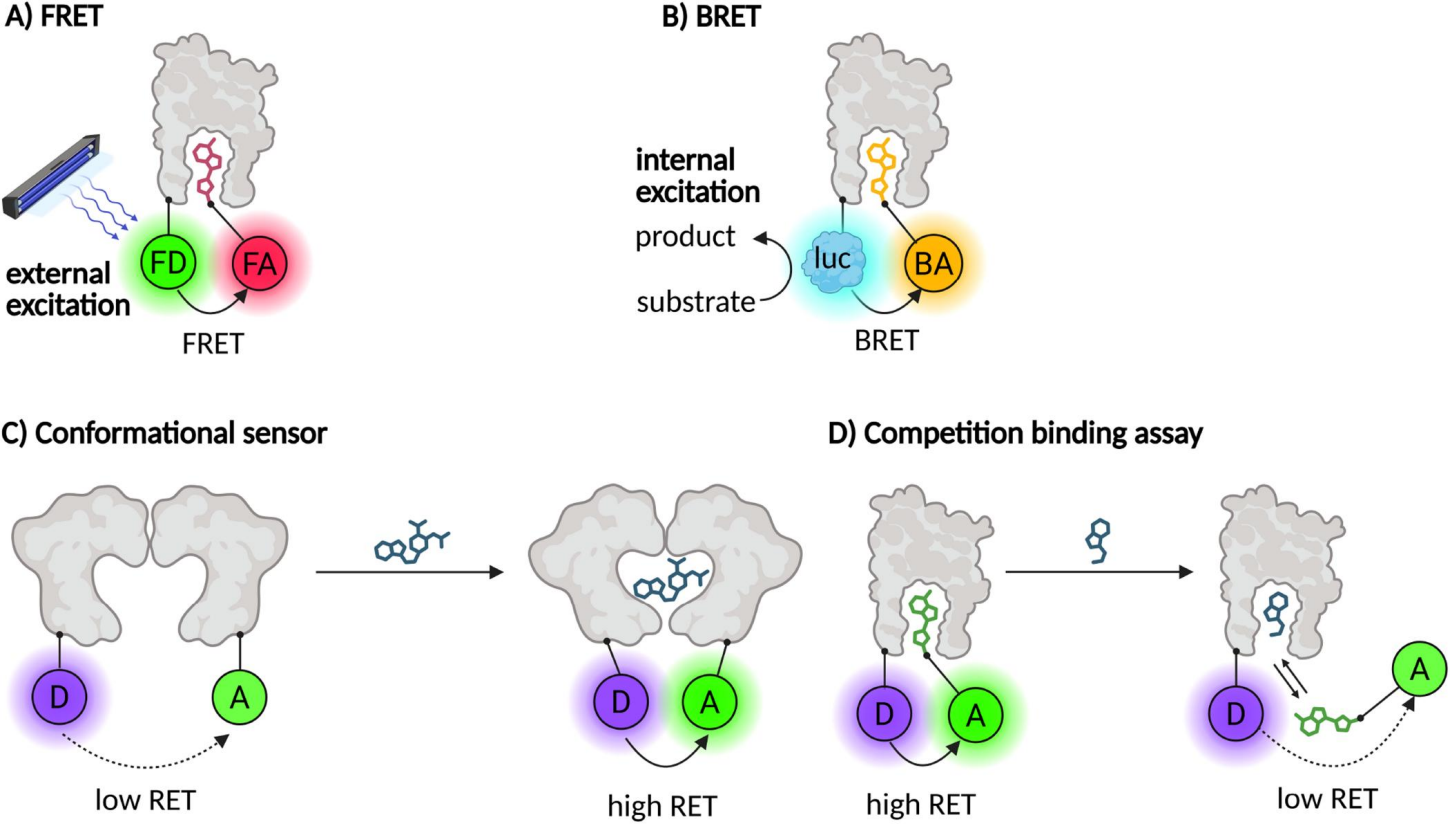
Schematic illustration of the assay principles of FRET and BRET as well as their application for ligand binding assays. (A) Schematic illustration of the FRET technology. (B) Schematic illustration of the BRET technology. (C) Schematic illustration of a FRET‐ or BRET‐based conformational sensor that can be used to study conformational changes of a target protein as a consequence of ligand binding. The low RET state is based on random interactions of donor and acceptor within the assay setup, whereas the high RET state is caused by a directed interaction of donor and acceptor due to a conformational change caused by ligand binding. (D) Schematic illustration of a FRET‐ or BRET‐based assay that enables monitoring ligand binding via competition with a fluorescent tracer. The high RET state is based on the binding of the fluorescent tracer, which causes the desired interaction between donor and acceptor. In contrast, the low RET state is based on random interactions of the displaced fluorescent tracer with a donor. FD, fluorescence donor, FA, fluorescence acceptor, BA, bioluminescence acceptor, D, fluorescence/bioluminescence donor, A, fluorescence/bioluminescence acceptor, RET, resonance energy transfer. Figure created in https://BioRender.com.

Compared with radioactive binding assays, proximity‐based techniques, such as Förster resonance energy transfer (FRET) and bioluminescence resonance energy transfer (BRET), the latter first described by Xu et al. in 1999 [41], can be performed using simple mix‐and‐measure protocols, enabling high‐throughput, plate reader‐based binding studies. In general, both FRET and BRET are based on resonance energy transfer between a donor and an acceptor, which requires (i) the spectral overlap between the emission spectrum of the donor and the excitation spectra of the acceptor, (ii) close proximity of these entities to each other, and (iii) depends on the relative orientation of the donor and acceptor transition dipoles (*i.e*., orientation factor). Figure 1 schematically illustrates the different principles of FRET‐ and BRET‐based assays as well as the application of these techniques for detecting ligand binding via conformational sensors or the competition with fluorescent tracers. A more detailed discussion of the physical principles of resonance energy transfer can be found elsewhere [42]. The fact that the proximity‐based principle of FRET and BRET does not require a removal of the unbound tracer fraction before assay readout makes these assays well suited for real‐time kinetic measurements and the detection of low‐affinity binders. The latter is particularly relevant for fragment‐based drug discovery (FBDD). Moreover, proximity‐based techniques enable cellular target engagement studies for intracellular target proteins and intracellular binding sites of membrane proteins [43, 44, 45, 46]. Whereas FRET requires external illumination for the excitation of a donor fluorophore, the BRET technique relies on bioluminescent enzymes (*i.e*., luciferases) as a donor, which are internally excited by the enzymatic conversion of specific substrates.

Over the years, BRET‐based methods have been progressively refined through the development of improved luciferases, substrates, and donor–acceptor pairs [34, 47]. The first‐generation BRET system, now referred to as BRET1, employed the 36 kDa *Renilla* luciferase (Rluc) as a donor, coelenterazine h as a substrate, and yellow fluorescent protein (YFP) as an acceptor [41]. In the second‐generation system, called BRET2, the spectral properties of the donor were shifted toward shorter wavelengths by using the Rluc mutant Rluc8 together with DeepBlueC (coelenterazine 400a) as the substrate [48]. This substantially improved the spectral separation between donor and acceptor emission, thereby reducing donor bleed‐through and background signal. Owing to their blue‐shifted donor emission, GFP2 and GFP10 served as the preferred acceptor fluorophores. Further improvements, including reduced cellular autofluorescence and more sustained light emission, were achieved with BRET3, which employed the 62 kDa firefly luciferase from *Photinus pyralis* (Fluc) with d‐luciferin as the substrate and cyanine‐labeled acceptor fluorophores [49, 50]. More recently, the significantly smaller (19 kDa) and substantially brighter (~150‐fold brighter than Rluc) NanoLuc luciferase (Nluc), used in combination with furimazine in so‐called NanoBRET assays, has become the preferred donor for BRET applications, owing to its superior brightness, stability, and spectral properties [51]. Due to its substantially improved brightness, Nluc does not necessarily require high overexpression, as compared with Rluc, to achieve adequate BRET signals. Further, the small size of Nluc facilitates the generation of fusion proteins while minimizing interference with the native structure and function of the target protein, an important advantage particularly for membrane proteins such as ion channels [52, 53]. As resonance energy acceptors, NanoBRET assays use small molecule‐based fluorescent tracers that target the protein of interest (POI) either by a direct ligand–protein interaction or by an indirect interaction mediated by an adapter protein (*i.e*., HaloTag) that is genetically fused to the POI [34, 54, 55].

Because BRET assays do not require external excitation (*e.g*., by a laser or lamp), they avoid donor excitation light bleed‐through into the acceptor detection channel, a major source of background in conventional FRET‐based assays. Moreover, the absence of external excitation minimizes photobleaching and cellular autofluorescence, thereby improving the signal‐to‐noise ratio and sensitivity. Consequently, BRET assays can offer substantially higher sensitivity than conventional FRET assays, with improvements of up to 100‐fold reported in some applications [32, 56]. It should be noted, however, that this particular advantage of BRET is less pronounced when compared with time‐resolved FRET (TR‐FRET) or lanthanide‐based FRET (LRET). In these approaches, the long fluorescence lifetimes of lanthanide donors allow delayed signal acquisition after short‐lived background fluorescence has decayed, thereby substantially reducing excitation‐related background and improving the signal‐to‐background ratio [57, 58]. However, these improved FRET techniques come along with the tradeoff of requiring specialized instrumentation enabling time‐resolved measurements, which may limit their accessibility and applicability in some experimental settings.

Due to their various aforementioned benefits, BRET assays have played a key role in drug discovery campaigns and have steadily increased in importance in recent years [33, 34, 59, 60, 61, 62, 63]. However, in contrast to other target classes (*e.g*., GPCRs, enzymes), where BRET‐based technologies are widely applied, they currently play only a minor role in the field of ion channels. This might be explained by the fact that the electrophysiological expertise in this field is very strong and patch‐clamp assays are still frequently used as primary assays for evaluating new ligands, despite their limitations mentioned above.

Herein, we summarize recent applications of BRET‐based methods for studying ligand binding to ion channels, highlighting both the challenges and the potential of this technology to advance drug discovery efforts for this target class.

## 2 BRET‐Based Approaches to Study Ligand‐Ion Channel Interactions

In contrast to BRET‐based approaches for studying ion channels, FRET‐based techniques have already been comprehensively reviewed in the recent literature [4, 57, 64]. Thus, we will focus in the following on BRET‐based applications for ion channels to address this gap in the current literature and stimulate further research and methodological development in this emerging field, while referring readers to existing reviews for comprehensive discussions of FRET‐based techniques.

In recent years, BRET‐based strategies have been applied across multiple channel families to investigate channel assembly [65, 66], direct steric interactions with GPCRs [67], and functional coupling between GPCRs and ion channels mediated via second messengers [68, 69]. While these applications demonstrate the broad utility of BRET‐based technologies for studying ion‐channel biology, they are conceptually distinct from approaches aimed at characterizing ligand‐ion channel interactions. In the following, we will primarily focus on the discussion of BRET‐based conformational biosensors (see Figure 1C), which enable to monitor ligand‐induced structural channel rearrangements, as well as BRET‐based ligand binding assays based on the displacement of small‐molecule fluorescent tracers (see Figure 1D), which directly quantify target engagement. For completeness, Supporting Information S1: Figure S1 provides an overview of the different conceptual approaches used in BRET‐based studies of ion channels reported to date.

### 2.1 BRET‐Based Conformational Biosensors

In the following, we discuss BRET‐based conformational biosensors that have been successfully developed for *K*_ir_/GIRK and TRPV channels, enabling real‐time monitoring of ligand‐induced structural rearrangements within ion channels in living cells. These approaches have also been adapted for multiplexed and high‐throughput applications, demonstrating their potential for comparative ion‐channel pharmacology and the identification of novel ion‐channel modulators.

#### 2.1.1 Bret‐Based Conformational Biosensors for K_ir_3 Channels

Already in 2015, Robertson et al. applied the concept of BRET‐based conformational biosensors to study ligand binding to K_ir_3 channels [69]. In general, G protein–gated inwardly rectifying potassium (K_ir_/GIRK) channels play a central role in regulating cellular excitability by coupling GPCR activation to membrane hyperpolarization and are implicated in a range of physiological and pathological processes, including cardiac arrhythmias [70] as well as neuronal functions such as pain modulation and addiction [71, 72]. Their pharmacological relevance is further underscored by clinically used drugs such as vernakalant, an antiarrhythmic agent approved by the European Medicines Agency (EMA) in 2010 [73]. Vernakalant acts as an atrial‐selective multichannel blocker, inhibiting several cardiac potassium currents, including the acetylcholine‐activated potassium current (I_KACh_) mediated by GIRK channels, while also exhibiting concentration‐, voltage‐, and frequency‐dependent blockade of sodium channels [73, 74]. K_ir_3 channels assemble as tetramers of different K_ir_3 subunits (K_ir_3.1–K_ir_3.4), each containing two transmembrane helices and a large cytoplasmic domain that forms the gating machinery [75]. Structural studies indicated that channel activation involves coordinated rearrangements of cytoplasmic and transmembrane gating elements, thus making these channels ideally suited for a proof‐of‐principle study to test the applicability of BRET‐based conformational biosensors to identify new drugs that influence ion‐channel function [76, 77]. As illustrated in Figure 1C, the measured BRET signal reflects ligand‐induced conformational rearrangements rather than direct target occupancy, an important distinction for the interpretation of conformational biosensors. To provide such a proof of principle, the authors engineered 10 different constructs, which were combined into 17 donor–acceptor BRET pairs. Donors Rluc or Nluc were fused to the distal C‐termini of K_ir_3.1 or K_ir_3.2 subunits, and the tetracysteine (TC)‐domain (CCPGCC) that binds the Fluorescein Arsenical Hairpin binder‐Ethanedithiol (FlAsH‐EDT2) acceptor was introduced at selected positions in the same or different subunits as the donor, so as to respectively yield intra‐ or intermolecular biosensors. The designed constructs were expressed in Human Embryonic Kidney‐293 Suspension Line (HEK293SL) cells. Among the investigated combinations, pairs bearing the donor Nluc at the C‐terminal end of K_ir_3.2 subunits and the FlAsH‐EDT2 acceptor at the N‐terminal end (NT) or the interfacial helix (N70) of K_ir_3.1 subunits were identified as the most promising conformational biosensors. These pairs displayed significant changes in energy transfer upon direct activation with K_ir_3 channel ligands (*e.g*., ML‐297, propranolol), but interestingly also via indirect stimulation of interacting GPCRs, such as the stimulation of the δ‐opioid receptor with deltorphin B. Moreover, the established conformational biosensors enabled cellular kinetic measurements in real time. The observed BRET changes associated with channel activation followed similar kinetics as the currents generated by activators, and pharmacological responses of conformational biosensors were in good agreement with known pharmacology of tested ligands. The fact that the K^+^ selectivity filter and the topology of the subunits that define the ion path are conserved across channels of increasing complexity, including voltage‐gated potassium (K_V_) channels [78], suggests that the conformational biosensors developed for K_ir_3 channels could be eventually used as templates for other related family members. However, as with other resonance energy transfer (RET)‐based approaches, the readout remains indirect and strongly dependent on sensor positioning, capturing only local structural changes. Moreover, as the developed conformational biosensor reports both direct channel activation by K_ir_3 agonists and indirect G‐protein mediated activation, it does not resolve which molecular step drives channel opening or whether additional factors contribute to the observed signal. Different activation mechanisms may therefore produce similar BRET signatures, limiting mechanistic interpretation. Despite these limitations, the ability to monitor conformational dynamics in real time within living cells provides clear added value over relying solely on functional assays.

#### 2.1.2 Multiplexed BRET Conformational Biosensors for TRPV Channels

In addition to K_ir_3, a second area of application for BRET‐based conformational sensors is the investigation of transient receptor potential vanilloid (TRPV) channels. TRPV channels are Ca^2+^‐permeable, nonselective cation channels that function as polymodal sensors in sensory and non‐sensory tissues. Within the transient receptor potential cation channel superfamily (TRPC, TRPA, TRPV, TRPM, TRPML, TRPP and TRPN), TRPV1, TRPV3, and TRPV4 are among the best characterized members. Structurally, TRPV channels assemble as tetramers, in which each subunit is formed by six transmembrane helices (S1–S6), intracellular N‐ and C‐termini, and a pore‐forming region between S5 and S6 [21, 79, 80, 81]. Structural and functional studies indicate that both termini contribute to channel regulation and gating through interactions with intracellular domains, including ankyrin repeats and the conserved C‐terminal TRP helix [21, 79, 80]. Given their roles in pain signaling, inflammation, pruritus and barrier disorders, TRPV channels are considered relevant therapeutic targets, further supported by the clinical use of the TRPV1 agonist capsaicin [80, 81]. Structural models further suggest close proximity and functional coupling between intracellular domains during gating transitions, supporting the feasibility of intramolecular conformational biosensor strategies [21, 79].

Key advances in this area were provided by Ruigrok and colleagues, who established a live‐cell BRET platform capable of resolving ligand‐ and temperature‐dependent conformational transitions of thermo‐TRP channels in real time [82]. Their assays were performed in transiently transfected HEK293T cells, enabling robust channel expression and kinetic measurements under intact regulatory cell conditions. In the intramolecular configuration, TRPV1 was engineered with the yellow fluorescent protein (YFP) fused to the N‐terminus, and Rluc fused to the C‐terminus, generating an intramolecular BRET biosensor in which conformational rearrangements within the tetrameric channel are reflected by changes in BRET efficiency using coelenterazine h as substrate. As each subunit carries both the donor and acceptor moieties, the measured signal likely arises from multiple intra‐ and inter‐subunit donor–acceptor configurations within the tetrameric channel complex. Using this construct, clear capsaicin‐ and heat‐dependent BRET responses were observed with rapid onset after stimulation, consistent with fast channel gating. Functional validation was achieved by stimulating TRPV1 with capsaicin or heat and comparing BRET responses with established functional activation profiles, confirming retention of native channel responsiveness.

**Figure 2:**
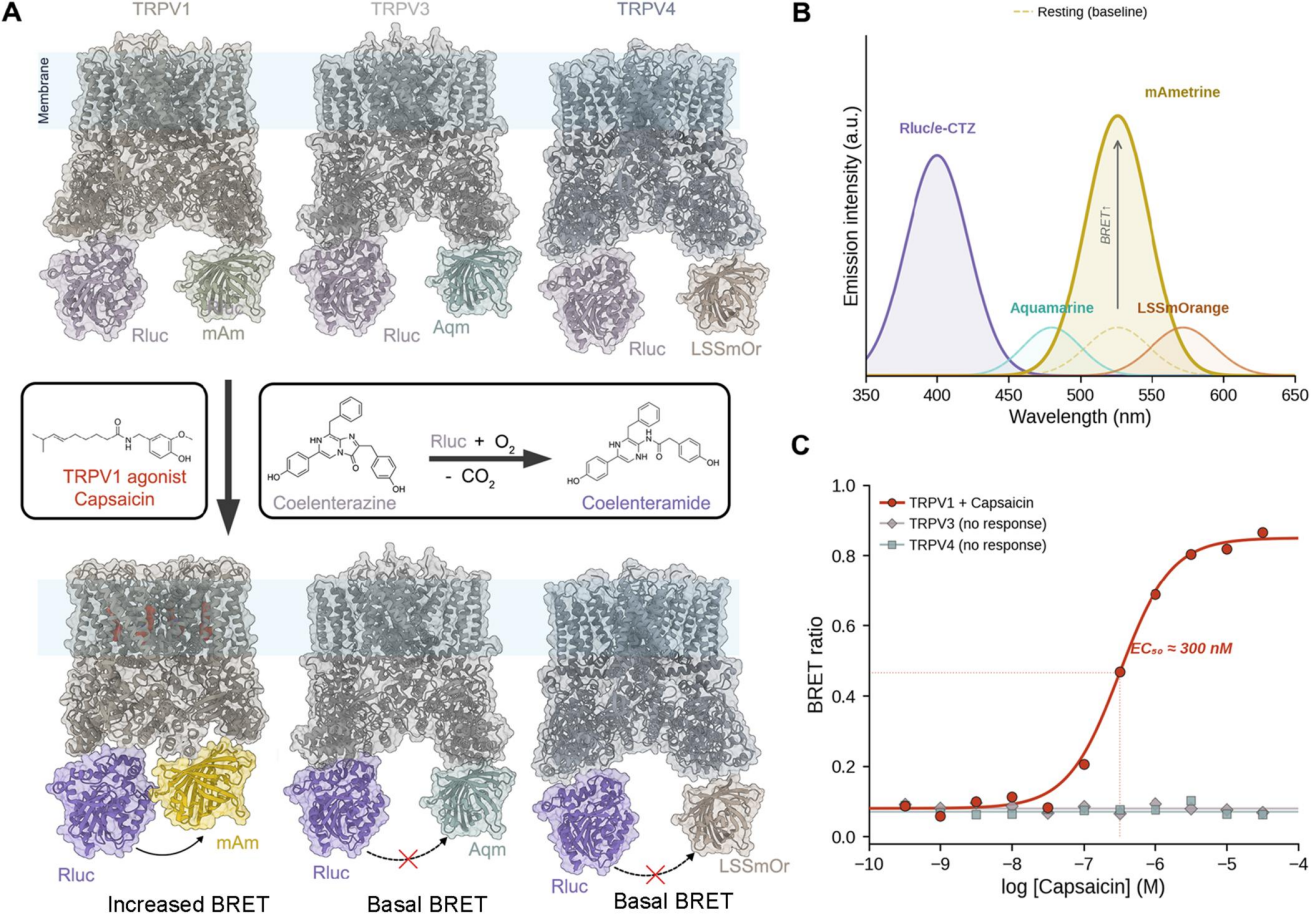
Schematic illustration of a multiplexed BRET‐based approach to monitor capsaicin‐induced activation of TRPV1, TRPV3, and TRPV4. (A) Design of TRPV–luciferase–fluorophore constructs and selective activation of TRPV1 by capsaicin as a TRPV1 agonist, using coelenterazine as a substrate for Rluc. (B) Emission spectra of the donor (Rluc) and acceptor fluorophores enabling multiplexed detection, demonstrating the selectivity of capsaicin for TRPV1 over TRPV3 and TRPV4. (C) Schematic concentration–response curve of capsaicin‐induced TRPV1 activation measured by BRET, showing no response for TRPV3 and TRPV4. Rluc: *Renilla* luciferase, Aqm, aquamarine; LSSmOr, large spectrum shift monomeric Orange; mAm, monomeric Ametrine; Rluc/e‐CTZ, *Renilla* luciferase enhanced coelenterazine. Figure created in https://BioRender.com.

Alongside intramolecular sensing, intermolecular assays utilizing YFP–calmodulin and TRPV1–Rluc constructs demonstrated activation‐dependent recruitment of calmodulin upon channel stimulation, linking conformational rearrangements to downstream regulatory interactions. An important aspect of the study was the development of a full‐spectral multiplex BRET strategy, which enables simultaneous monitoring of TRPV1, TRPV3, and TRPV4 within a single sample. Multiplexing was achieved using spectrally engineered fluorescent proteins with large Stokes shifts (*e.g*., aquamarine, mAmetrine, and LSSmOrange) in combination with luciferase donors, enabling full spectral deconvolution of overlapping BRET signals (see Figure 2).

This technique allows parallel deconvolution of channel‐specific responses under identical experimental conditions. Importantly, this approach provides a powerful and scalable platform for high‐throughput screening of ligands across closely related TRPV channels, which share substantial structural similarity. Moreover, it offers the possibility to assess off‐target effects within a single cellular system in real time, thereby improving comparability between measurements while reducing experimental variability, well‐to‐well bias and material consumption. In multiplexing strategies, BRET offers distinct advantages over FRET‐based binding assays, as the absence of external excitation minimizes autofluorescence and reduces spectral crosstalk between signals and channels in parallel detection setups. These limitations become particularly critical in multiplexed settings, where signal overlap and background contributions can accumulate across multiple reporters. In the experiments performed by Ruigrok et al., TRPV3 and TRPV4 were analyzed using analogous calmodulin‐recruitment configurations, revealing subtype‐specific activation profiles and demonstrating the robustness of the platform for comparative thermo‐TRP pharmacology. Ruigrok and colleagues further emphasized that biosensor topology critically determines assay performance. Terminal fusions caused minimal disruption to the transmembrane core and preserved intracellular regulatory interactions. Prior ion‐channel biosensor studies also show that donor–acceptor placement strongly influences signal amplitude and interpretation [21, 79, 80]. Beyond qualitative observations, the authors provided robust quantitative pharmacological readouts, with favorable Z' factors (0.6–0.7) supporting the suitability for HTS due to high signal separation and reproducibility in live cells. Time‐resolved measurements further enable characterization of TRPV activation dynamics, with capsaicin‐induced conformational changes occurring on the second timescale (τ ≈ 2 s). Distinct kinetic signatures were observed for chemical and thermal stimulation, consistent with multiple conformational transitions during channel gating. Despite these advances, several considerations should be noted. As with other RET‐based approaches, BRET reports conformational dynamics through changes in donor–acceptor proximity and orientation rather than discrete structural states. In addition, direct conformational readouts were primarily established for TRPV1, whereas TRPV3 and TRPV4 were mainly assessed via calmodulin recruitment, limiting direct subtype comparisons. Pharmacological validation was largely performed using established reference agonists and antagonists, and broader ligand coverage remains to be explored.

#### 2.1.3 High‐Throughput Screening Applications of TRPV1 BRET Biosensors

To evaluate whether conformational BRET‐based biosensors can be implemented in drug discovery workflows, Chappe et al. adapted the previously established TRPV1 intra‐ and intermolecular probes by Ruigrok et al. to an HTS format and benchmarked them against an automated calcium assay (ACA) and APC [82, 83]. Functional validation was performed in HEK293 cells transiently expressing either the intramolecular TRPV1 construct, in which YFP was fused to the N‐terminus and Rluc to the C‐terminus, or the intermolecular configuration combining TRPV1–Rluc with YFP‐tagged calmodulin. To verify whether both BRET constructs are functional, HEK293 cells were stimulated with increasing concentrations of established TRPV1 agonists (*e.g*., capsaicin, resiniferatoxin), resulting in a concentration‐dependent increase in BRET signals consistent with published findings.

Antagonist sensitivity was confirmed using six reference inhibitors (*e.g*., CPZ, AMG519), which suppressed capsaicin‐induced activation in both assays in agreement with prior studies. Potency estimates for the tested ligands were comparable across BRET, APC, and ACA platforms. Z' values of ~0.5–0.6 were consistently observed across independent HTS runs and replicate analyses. The platform was subsequently applied to screen the Prestwick Chemical Library (1200 compounds), enabling the identification of more than 50 novel TRPV1 ligands. These primary hits were grouped into 11 distinct chemotype clusters, encompassing both activating and inhibitory compounds. Notably, compounds within individual clusters exhibited consistent activity profiles across assays, suggesting that structurally related chemotypes share common functional and potentially mechanistic properties in modulating TRPV1.

#### 2.1.4 Conformational Biosensors for Studying Local Ion‐Channel Microenvironment

Beyond conformational readouts that enable to monitor structural changes within ion‐channel architecture, genetically encoded BRET probes have also emerged as valuable tools to investigate the local ion‐channel microenvironment. A recent study by Chappe et al. [84] reported ion‐sensitive conformational BRET‐based biosensors that were intracellularly attached to certain ion channels, to monitor changes in ion concentrations in direct proximity to the intracellular opening of the channel pore (see Supporting Information S1: Figure S1), thereby representing an important emerging application of genetically encoded BRET probes in ion‐channel pharmacology. In particular, this technology enabled real‐time monitoring of changes in Ca^2+^, K^+^ concentrations and ionic strength in the nanoenvironment of TRPV1 and P2X5/P2X7 pores in response to agonist or antagonist addition. These measurements revealed ligand bias‐dependent cationic selectivity and suggested competition between different cations for access to the channel pore. The findings by Chappe et al. further highlight the versatility of BRET‐based conformational biosensors for studying ion‐channel function beyond direct ligand‐binding measurements.

### 2.2 BRET‐Based Binding Assays Using Fluorescent Tracers

In addition to BRET‐based biosensors for monitoring ligand‐induced conformational changes of ion channels, a few BRET‐based binding assays using fluorescent small molecule tracers have already been reported. Unlike the conformational biosensors discussed above, these assays directly quantify ligand binding through fluorescent tracer displacement and thus provide a direct measure of target engagement, as schematically illustrated in Figure 1D.

#### 2.2.1 NanoBRET Binding Assay for TRPML

In 2023, Cunha et al. described a NanoBRET‐based binding assay for the transient receptor potential mucolipin 1 (TRPML1) enabling determination of equilibrium constants and kinetic parameters of unlabeled ligands in intact HEK293T cells [55]. TRPML1 is a homotetramer encoded by the gene *Mucolipin‐1* (*MCOLN1)* [85]. Each subunit contains four distinct structural features: a membrane‐spanning segment composed of six transmembrane helices, cytosolic C‐ and N‐termini, two pore helices and a luminal domain [86]. In the human body, TRPML1 is responsible for the release of Ca^2+^ ions from lysosomes and for the regulation of endolysosomal trafficking and phagocytosis [87]. Accordingly, mutations in *MCOLN1*, which result in an altered activity of TRPML1 variants, affect autophagy and Ca^2+^ homeostasis, and are associated with the formation of several diseases. The most common conditions linked to dysfunctional TRPML1 variants include lysosomal storage disorders (*e.g*., mucolipidosis type IV), neurodegenerative diseases, and cancer [86, 87, 88, 89]. Consequently, this ion channel has gained interest as a therapeutic target to treat these conditions. To facilitate a high‐throughput screening of existing libraries, the authors established a NanoBRET‐based binding assay [55].

**Figure 3:**
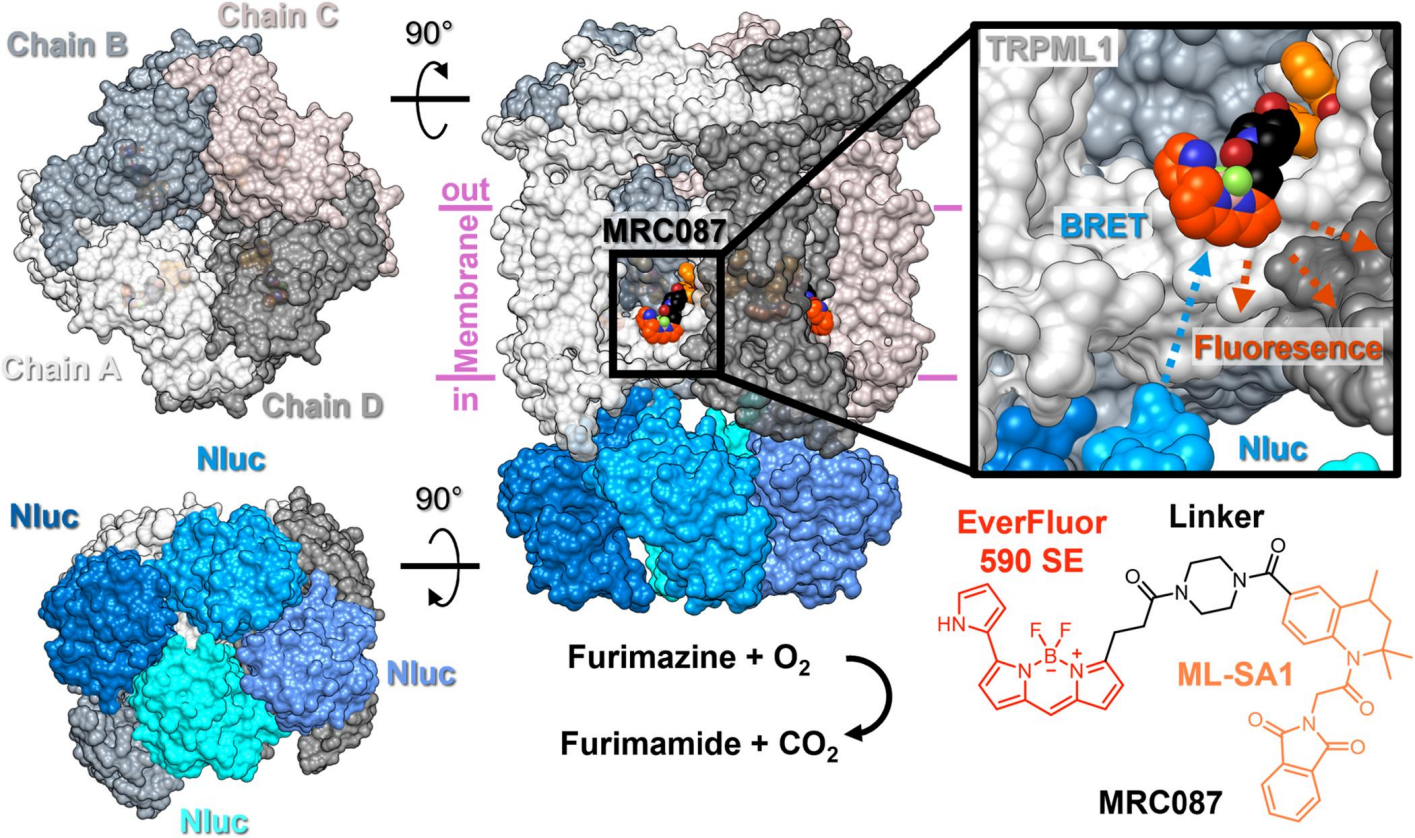
Schematic fusion of Nluc (PDB ID 7SNS, blue and green surfaces) to the C‐terminus of human TRPML1 (gray surfaces, PDB ID 9HL3) [89]. All four TRPML1 subunits and four Nluc molecules are depicted. In the cryo‐EM structure, TRPML1 is bound to the activator ML‐SA1 (orange), which is part of the fluorescent probe MRC087. MRC087 consists of ML‐SA1 attached to EverFluor 590 SE (red) via a linker (black). The zoomed‐in binding mode of MRC087 illustrates how it acts as a BRET acceptor. The protein subunits and the binding mode of the probe were modeled based on the experimental structure in the molecular operating environment (MOE) and visualized with UCSF Chimera.

This assay uses Nluc as a BRET donor with furimazine as a substrate, and the well‐characterized synthetic TRPML1 agonist ML‐SA1 labeled with the fluorophore EverFluor 590 SE to generate the fluorescent tracer MRC087, which serves as the BRET acceptor [55] Since cryo‐EM structures of TRPML1 in complex with ML‐SA1 revealed that the ligand binding site is located in close proximity to the cytosolic side of the ion channel (PDB ID 9HL3) [89], Nluc was fused to the cytosolic C‐terminus of TRPML1 by a flexible linker, consisting of nine amino acid residues, to achieve the desired BRET (see Figure 3).

Before testing the NanoBRET system on its functionality, the BRET donor and acceptor were characterized separately. To evaluate cell permeability of the fluorescent probe, which was crucial for reaching its binding site at the cytosolic face of the channel, and its colocalization with TRPML1, the authors used HEK293T cells expressing TRPML1 fused to YFP. Fluorescence microscopy showed that the fluorescent probe penetrates the cell membrane and accumulates intracellularly. In addition, TRPML1 fused to Nluc was expressed independently in HEK293T cells and exhibited robust luminescence emission. Following the characterization of the donor and acceptor, the interaction between them was assessed by titrating TRPML1‐expressing HEK293T cells with the fluorescent probe and measuring the BRET signal, which resulted in a saturation curve. To confirm that the BRET signal resulted from specific binding of the fluorescent probe to TRPML1, a known non‐fluorescent ligand was added at a saturation concentration and was able to displace the fluorescent probe from its binding site, which resulted in a strongly reduced BRET response. From these data, the authors determined the binding affinity (K_d_) of the fluorescent probe and calculated the Z'‐factor of > 0.5, indicating that this assay is suitable for HTS approaches. To further assess the applicability of this assay for studying the binding of unlabeled ligands, a competition binding experiment was performed using one TRPML1 agonist and one antagonist, respectively. The resulting concentration–response curves from these competition binding assays led to apparent equilibrium dissociation constants (K_dA_) that were consistent with EC_50_ or IC_50_ values obtained from a Ca^2+^ indicator assay.

In addition to measurements under equilibrium conditions, the authors demonstrated the suitability of their BRET‐based assay not only for evaluating kinetic parameters (*i.e., k*_on_ (association rate constant) and *k*_off_ (dissociation rate constant)) for the fluorescent probe, but also for competing agonists and antagonists, respectively. For the fluorescent probe, the data obtained from real‐time kinetic measurements in live HEK293T cells enabled calculation of a kinetic *K*_d_ value, which was consistent with the *K*_d_ value obtained under equilibrium conditions. To study the kinetic binding of non‐fluorescent ligands, kinetic competition binding assays were performed, where fixed concentrations of the fluorescent probe were co‐incubated with varying concentrations of non‐fluorescent competitors. The specific BRET signals that were detected over time were fitted with the Motulsky–Mahan competitive binding model and allowed the estimation of the corresponding *k*_on_ and *k*_off_ values of the unlabeled ligands. Consequently, the designed NanoBRET‐based assay platform enables the determination of binding affinities and kinetic parameters of unlabeled ligands for TRPML1. However, this assay has thus far only been validated with a single known agonist and a single known antagonist, and its broader applicability remains to be demonstrated.

#### 2.2.2 NanoBRET Assays for Lipid‐Potassium Channel Interactions

Given that certain ion channels are strongly regulated by low‐abundance signaling lipids such as phosphatidylinositol 4,5‐bisphosphate (PIP_2_), and the underlying regulation mechanisms are not yet fully understood, Cabanos et al. used a NanoBRET approach to examine the lipid regulation of different potassium channels, including Tandem of P‐domains in a Weakly Inward rectifying K^+^ channel 1 (TWIK‐1)‐related K^+^ channel (TREK‐1), TWIK‐related arachidonic acid activated K^+^ channel (TRAAK), and inwardly rectifying potassium (K_ir_) channels 2.1 and 2.2 [32] TREK‐1 and TRAAK are mechanosensitive ion channels belonging to the two‐pore domain potassium channel family. Both channels are mainly expressed in the nervous system and responsible for maintaining resting potential and modulating neuronal excitability [90, 91, 92, 93, 94]. Dysregulation of TRAAK is associated with several diseases such as chronic pain, epilepsy, and developmental disorders [95, 96]. In contrast, TREK‐1 dysfunction is linked to depression, Alzheimer's, and cardiac ischemia [97, 98, 99] K_ir_2.1 and K_ir_2.2 share the involvement in maintaining resting potential and cellular excitability with TREK‐1 and TRAAK channels [100, 101].

For the identification of new lipid‐based interaction partners of TREK‐1, TRAAK, K_ir_2.1, and K_ir_2.2 that bind to the same binding site as PIP_2_, Cabanos et al. developed a NanoBRET competition binding assay. To this end, fluorescently labeled phosphatidylinositol‐4,5‐bisphosphat (FL‐PIP_2_) bearing a BODIPY‐TMR‐based fluorophore was used as a BRET acceptor. Since the PIP_2_ binding site is located within the transmembrane domain near the intracellular face of the ion channel, all investigated ion channels were engineered with Nluc fused to their intracellular C‐termini in close proximity to the PIP_2_ binding site [32]. In contrast to the other BRET approaches discussed in this review, Cabanos et al. employed a cell‐free setup, likely to circumvent issues related to the limited membrane permeability of PIP_2_ and its fluorescent analog. The purified potassium channels were obtained by expression in yeast (*Pichia pastoris*), detergent extraction, and a two‐step purification by affinity and size exclusion chromatography.

In first experiments, the authors showed that FL‐PIP_2_ binds to TREK‐1, TRAAK, K_ir_2.1, and K_ir_2.2 and can be displaced by high concentrations of the non‐fluorescent PIP_2_, thus indicating specific binding of the fluorescent tracer. In concentration‐dependent competition binding studies, the authors showed that PIP_2_ binds to the studied potassium channels with affinities in the low micromolar to sub‐micromolar range, with the highest affinity for K_ir_2.2 (*K*_d_ = 0.1 µM). The affinities detected for phosphatidic acid (PA) were about one order of magnitude weaker, but still indicated robust binding. Next, the NanoBRET assay was used to screen a small library of soluble short‐chain and native membrane lipids for binding to TREK‐1. While PIP_2_ and phosphatidic acid (PA) showed the strongest binding, several additional lipids, including phosphatidylglycerol (PG) as well as ω‐3 and ω‐6 arachidonic acids, were also identified as new binders. Using a cell‐free reconstituted system for functional ion flux assays, the newly identified TREK‐1 binders PA and PG were further characterized, showing a modulation of ion flux. Whether the observed agonistic effects of these lipids translate into pharmacological agonism in a native membrane context, where lipid composition, membrane tension, and auxiliary proteins may all contribute to channel activity, remains to be established.

The comparison of the two NanoBRET‐based competition binding assays discussed above highlights the versatility of these approaches for studying ion channels, ranging from endpoint measurements with purified proteins to real‐time kinetic analyses in whole‐cell systems. In both cases, constructs were designed with Nluc fused to the intracellular C‐terminus of the respective ion channel, and fluorescent tracers targeting binding sites in close proximity to the intracellular channel surface. This setup offers a promising perspective for extending such NanoBRET‐based approaches to other ion channels with ligand‐binding sites accessible from the cytosol, including voltage‐gated sodium channels (*e.g*., Na_v_1.8) and adenosine triphosphate (ATP)‐sensitive potassium channels (K_ATP_). However, a major limitation of these methods remains the limited availability of suitable fluorescent tracers, as well as the labor‐intensive design and implementation of appropriate fusion constructs.

In summary, the BRET‐based binding assays using fluorescent tracers discussed in this subchapter and the BRET‐based conformational sensors discussed above represent two complementary strategies for investigating ion channels (see Figure 1). Together, these approaches enable the study of ligand‐induced conformational rearrangements and local channel microenvironments, as well as the direct quantification of target engagement, characterization of binding kinetics, and identification of specific binding sites and mechanisms of action, highlighting their considerable potential for advancing ion channel‐focused drug discovery.

## 3 Conclusion and Outlook

Despite considerable progress in BRET‐based technologies for studying ligand–target protein interactions, and the steadily increasing number of applications for other therapeutically highly relevant drug target classes (*e.g*., GPCRs, enzymes) in recent years, BRET still plays a minor role in the field of ion channels. This may be attributed to the strong electrophysiological expertise in the field and the continued use of patch‐clamp assays as primary tools for ligand evaluation, despite their limitations, particularly with respect to throughput. Nevertheless, the limited but emerging literature on BRET‐based approaches for studying ligand–ion channel interactions underscores the potential of this technique to deepen our understanding of ion‐channel function and to support drug discovery efforts in this area.

Many of the advantages of the BRET technology, including its suitability for HTS approaches and kinetic measurements in real time, its versatility ranging from isolated ion channels to whole‐cell setups, and its compatibility with multiplexing strategies for selectivity profiling, have already been demonstrated for individual ion channels.

With respect to the suitability for HTS, both BRET‐based conformational sensors for TRPV1 and competition binding assays for TRPML1 have been characterized with favorable Z' factors (> 0.5), thus indicating the applicability of these approaches to screen larger compound libraries [55, 82, 83]. However, it should be noted that only the study by Chappe et al. has actually applied the respective BRET‐based technology in a full HTS campaign, which resulted in the identification of new scaffolds for TRPV1 activators and inhibitors, providing a foundation for future drug discovery efforts [83].

As the determination of kinetic parameters becomes increasingly important in drug discovery—given that in vivo efficacy and clinical success of drug candidates often depend on drug–target residence time (RT) [30, 102], defined as the reciprocal of the dissociation rate constant k_off_ (RT = 1/k_off_)—the importance of BRET‐based approaches that enable real‐time kinetic measurements is expected to grow. The reported kinetic ligand binding studies on K_ir_3, TRPV1, and TRPML1 clearly demonstrate that BRET‐based techniques allow the determination of these key parameters for ion channel‐targeted ligands, both with conformational sensors and with competition binding assays based on fluorescent tracers.

Although still limited in number, the available literature already reflects the high versatility of BRET‐based binding assays for ion channel‐targeted applications, ranging from straight forward endpoint measurements with purified proteins to more complex real‐time kinetic competition binding assays in whole cells. Notably, BRET‐based competition binding assays employing fluorescent tracers have so far only been conducted with intracellularly Nluc‐labeled ion channels, thus only allowing the use of fluorescent tracers binding to regions proximal to the intracellular face of the channel. Future applications for monitoring extracellular binding should, however, be possible by inserting an Nluc into an extracellular loop of the respective channel, in a similar manner as this has been established for GPCRs [103].

Further, the manuscript by Ruigrok et al. on TRPV channels highlights the suitability of ion channel‐targeted BRET approaches for full‐spectral multiplex strategies, enabling simultaneous assessment of ligand binding to several ion channels within a single experiment [82]. Such multiplexing approaches may facilitate early detection of off‐target effects across closely related ion‐channel subtypes, which is especially relevant given the high structural similarity within many channel families.

However, in addition to their undeniable advantages, BRET‐based approaches for detecting ligand binding to ion channels are also associated with certain limitations and challenges.

First, they rely on artificial, genetically encoded fusion constructs, which may interfere with native channel behavior and require careful optimization of biosensor topology. Unlike predominantly monomeric GPCRs, ion channels typically assemble into homo‐ or heteromeric complexes, posing specific challenges for the design of suitable BRET constructs. In homomeric channels, such as the homotetrameric TRPML1 [55], each subunit may carry a luciferase tag, resulting in four donor moieties per channel complex with potentially variable spatial arrangements and substantially increasing the molecular bulk attached to the channel. Such modifications may sterically affect channel assembly or interfere with the native structure and function of the channel. For heteromeric channels, such as K_ir_3.1/3.2, the situation is further complicated by the need to control subunit stoichiometry and the incorporation of tagged versus untagged subunits. These challenges may be exacerbated for luciferases such as Rluc, which generally require higher expression levels to generate sufficient BRET signals. Overexpression of tagged subunits may promote non‐stoichiometric incorporation and result in heterogeneous populations of channel complexes containing different numbers of tagged subunits. Consequently, the measured BRET signal may represent a composite of multiple channel populations and donor–acceptor configurations, potentially complicating mechanistic interpretation. This represents an important ion channel‐specific challenge that distinguishes these targets from predominantly monomeric proteins such as GPCRs and many enzymes and further underscores the advantages of Nluc‐based systems, which can generate robust BRET signals at substantially lower expression levels.

Second, although the study by Ruigrok et al. on TRPV channels demonstrates the suitability of ion channel–targeted BRET approaches for multiplexing strategies [82], the multiplexing capabilities of BRET assays remain more limited than those of FRET‐based techniques.

This limitation primarily arises from the lower signal intensities of BRET compared with what is achievable with the direct illumination of FRET‐based approaches. Consequently, BRET generally requires the use of emission filters with broader bandwidths to maintain sufficient signal intensity. In contrast, the higher signal intensities achievable with FRET allow narrower emission filters to be employed, thereby providing greater spectral separation and facilitating higher‐order multiplexing as compared with BRET.

Third, the requirement for substrate addition may also impose limitations on BRET‐based kinetic measurements. In particular, for the characterization of very rapid association and dissociation kinetics, techniques based on rapid external excitation by a laser or lamp, such as FRET, may offer advantages because they enable continuous signal acquisition without the need for manual substrate addition, which typically introduces a delay of at least several seconds.

Fourth, standard BRET systems generally do not provide the single‐molecule resolution that can be achieved with other techniques such as single‐molecule FRET (smFRET) or single‐molecule fluorescence ligand binding (smFLiB) [104]. Such single‐molecule approaches are particularly valuable for resolving rare conformational states [46, 105], distinguishing between mechanisms that may be indistinguishable at an ensemble level [106], and have already been established for ion channels and related membrane transport proteins such as ABC transporters [45].

Fifth, the development of suitable fluorescent tracers for BRET applications remains a time‐ and labor‐intensive process, and suitable tracers are currently available for only a limited number of ion‐channel targets, thereby representing one of the main practical bottlenecks for a broader adoption of BRET in ion‐channel research. The major challenges in tracer development include: (i) identifying a suitable site for fluorophore attachment, which can be particularly challenging in the absence of structural information on the ligand–target complex or established structure–activity relationship (SAR) data; (ii) synthesizing the corresponding fluorescent conjugate, although this step can often be streamlined by employing robust and high‐yielding methods of modern click chemistry; (iii) confirming that the conjugate retains the desired binding mode and determining its affinity; (iv) establishing adequate cell permeability, which is particularly important when targeting intracellular binding sites; and (v) optimizing the linker and fluorophore to achieve a suitable balance between affinity, cellular permeability, and BRET signal intensity. Despite these challenges, the successful and increasingly broad application of BRET‐based binding assays across related target classes, such as GPCRs, demonstrates that systematic tracer development strategies can overcome many of these limitations.

Sixth, because BRET‐based ligand‐binding assays primarily capture local proximity changes upon ligand binding, they provide only limited mechanistic insight. In the case of BRET‐based conformational sensors, distinct activation mechanisms may therefore produce similar BRET signatures. Likewise, in competition binding assays, both activators and channel blockers can yield comparable competition profiles if they bind to the same site as the fluorescent tracer, thereby limiting mechanistic interpretation.

Therefore, BRET‐based assays for studying ligand binding to ion channels are particularly valuable when complemented with functional assays such as electrophysiological methods (*e.g*., patch‐clamp). Ultimately, integrating binding, functional, and multiplexed readouts across complementary and orthogonal assay formats will provide a more comprehensive and mechanistically informed view of ion‐channel pharmacology and significantly advance drug discovery efforts for this therapeutically important target class. With regard to the potential expansion of BRET‐based binding assays for ion channels, the numerous posters from different research groups presented on this topic at the Biophysical Society Annual Meeting 2026 (BPS2026) [107, 108, 109], together with published technical reports, point toward increasing methodological development in this space and support an overall optimistic outlook for the future.

## Conflicts of Interest

The authors declare no conflicts of interest.

## Supporting information


Supporting File

## Data Availability

Data sharing is not applicable to this article as no datasets were generated or analyzed during the current study.
