# Supplementary material for: BRET‐Based Approaches for Ion Channels: Emerging Applications and Future Directions
Source: Arch Pharm (Weinheim). 2026 Sep 3;359(9):e70325. doi: 10.1002/ardp.70325 (PMC13540534; doi:10.1002/ardp.70325)
Supplement: Supplementary file 1 [file ARDP-359-e70325-s001.pdf]

Supporting Information

BRET-based approaches for ion channels: emerging applications and future directions

Nadine Kock[1,2#], Laura Heitzer[1#], Joana Massa[1,2], Marvin Taterra[1,2], Marcel Bermúdez[1,2], Oliver Koch[1,2], Matthias Schiedel[1,2,*]

1 Institute for Pharmaceutical and Medical Chemistry, Faculty of Chemistry and Pharmacy, University of Münster, Münster, Correnssstraße 48, D-48149 Münster, Germany
2 GRK 2515, Chemical biology of ion channels (Chembion), Universität Münster, Correnssstr. 48, D-48149 Münster, Germany

[#]equal contribution
*Correspondence: Prof. Dr. Matthias Schiedel, Institute for Pharmaceutical and Medical Chemistry, Faculty of Chemistry and Pharmacy, University of Münster, Münster, Correnssstraße 48, D-48149 Münster, Germany
Email: matthias.schiedel@uni-muenster.de

Table of Contents

Page	Contents
S3	Supplementary Figure
S4	Supplementary References

SUPPLEMENTARY FIGURE

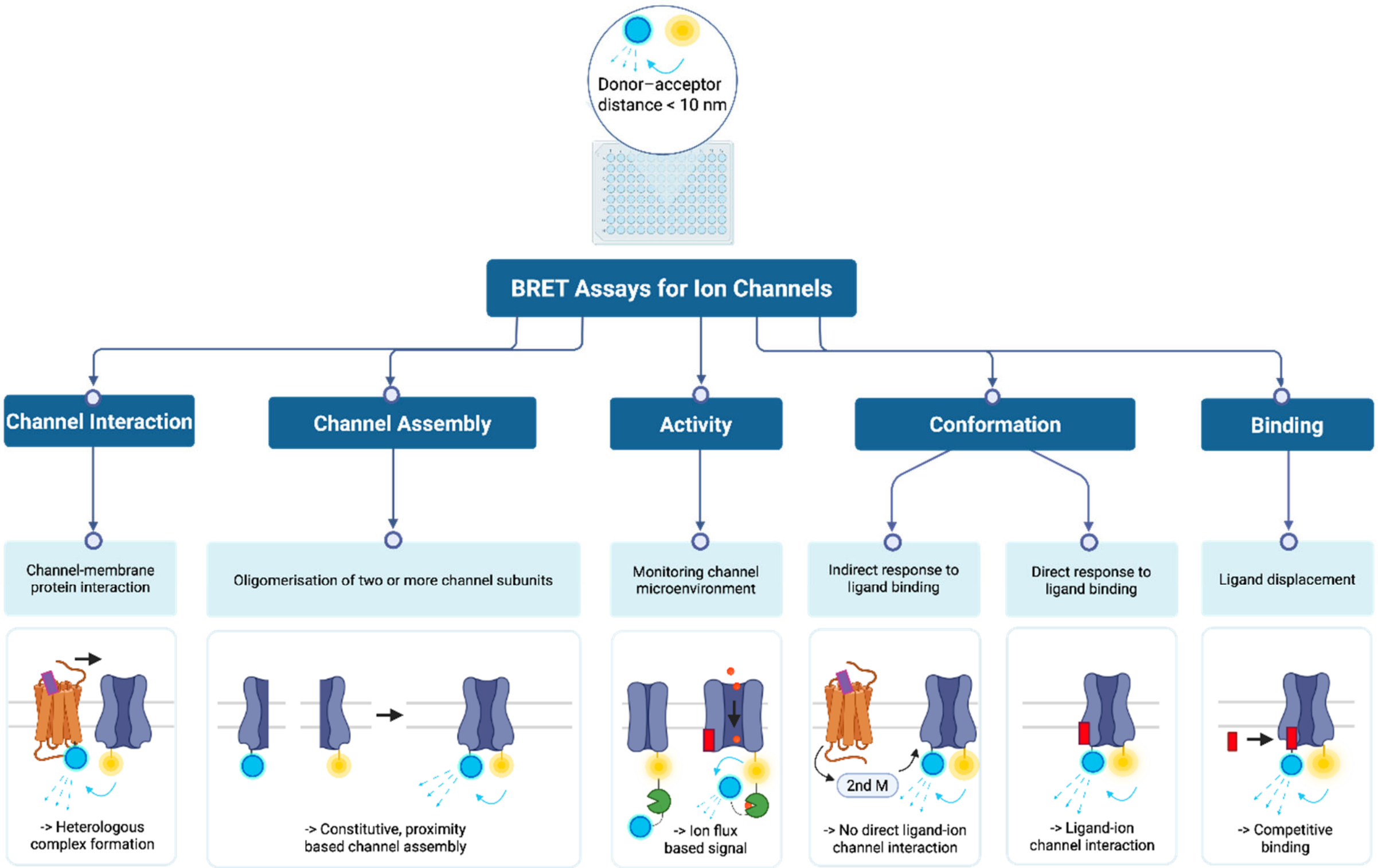

Figure S1: Schematic overview of the different conceptual approaches used in BRET-based studies of ion channels reported to date, ranging from the detection of protein-protein interactions between ion channels and other membrane proteins (*i.e.,* GPCRs),[1] channel assembly,[2,3] channel microenvironment,[4] indirect interactions between ion channels and GPCRs via second messenger-induced conformational changes in ion-channel structure,[5,6] conformational changes of ion-channel structure induced by a direct ligand-ion channel interaction,[6,7] and ligand binding by displacement of a fluorescently labelled ion-channel ligand.[8,9] Red dots represent ions that are passing the open pore of an ion channel. Red rectangles represent small-molecule ion-channel ligands. Violet rectangles represent small-molecule GPCR ligands. Yellow spheres are representing the BRET donors, light blue spheres the BRET acceptors. 2nd M denotes a second messenger. The green circular sector represents an ion-sensitive and ion-selective conformational sensor engineered between a BRET donor and acceptor. Figure created in https://BioRender.com.

SUPPLEMENTARY REFERENCES

[1] H. Tong, A. J. Gibb, *J. Physiol.* **2008**, *586*, 4693, DOI 10.1113/jphysiol.2008.158931.

[2] G. M. Whitaker, E. A. Accili, *Methods Mol. Biol. (Clifton, N.J.)* **2008**, *491*, 189, DOI 10.1007/978-1-59745-526-8_15.

[3] R. Flynn, K. Chapman, M. Iftinca, R. Aboushousha, D. Varela, C. Altier, *J. Biol. Chem.* **2014**, *289*, 16675, DOI 10.1074/jbc.M114.558668.

[4] Y. L. Chappe, S. Pierredon, A. Joushomme, P. Molle, A. Garenne, A. Canovi, S. Barbeau, F. Poulletier De Gannes, A. Hurtier, I. Lagroye et al., *Proc. Natl. Acad. Sci. U. S. A.* **2022**, *119*, e2205207119, DOI 10.1073/pnas.2205207119.

[5] C. Pétigny, A.-A. Dumont, H. Giguère, A. Collette, B. J. Holleran, M. Iftinca, C. Altier, É. Besserer-Offroy, M. Auger-Messier, R. Leduc, *Int. J. Mol. Sci.* **2022**, *23*, 2502, DOI 10.3390/ijms23052502.

[6] D. N. Robertson, R. Sleno, K. Nagi, D. Pétrin, T. E. Hébert, G. Pineyro, *Methods* **2016**, *92*, 19, DOI 10.1016/j.ymeth.2015.07.011.

[7] H. J. Ruigrok, G. Shahid, B. Goudeau, F. Poulletier De Gannes, E. Poque-Haro, A. Hurtier, I. Lagroye, P. Vacher, S. Arbault, N. Sojic et al., *Biophys. J.* **2017**, *112*, 87, DOI 10.1016/j.bpj.2016.11.3197.

[8] M. R. Cunha, C. M. C. Catta-Preta, J. E. Takarada, G. A. Moreira, K. B. Massirer, R. M. Couñago, *J. Biol. Chem.* **2023**, *299*, 104807, DOI 10.1016/j.jbc.2023.104807.

[9] C. Cabanos, M. Wang, X. Han, S. B. Hansen, *Cell Rep.* **2017**, *20*, 1287, DOI 10.1016/j.celrep.2017.07.034.
